# Emerging mercury and methylmercury contamination from new artisanal and small-scale gold mining along the Nile Valley, Egypt

**DOI:** 10.1007/s11356-023-25895-9

**Published:** 2023-02-25

**Authors:** Ahmed Abdelaal, Mohamed Sultan, Abotalib Z. Abotalib, Mohamed Bedair, R. V. Krishnamurthy, Mohamed Elhebiry

**Affiliations:** 1 Environmental Sciences Department, Faculty of Science, Port Said University, Port Said, 42526 Egypt; 2 Department of Geological and Environmental Sciences, Earth Sciences Remote Sensing (ESRS) Facility, Western Michigan University, Kalamazoo, MI 49008 USA; 3 Department of Geology, National Authority for Remote Sensing and Space Sciences, Cairo, 1564 Egypt; 4 Viterbi School of Engineering, University of Southern California, Los Angeles, CA USA; 5 Sukari Gold Mine, Centamin PLC, 85831 Egypt; 6 Geology Department, Faculty of Science, Al-Azhar University, Cairo, 11884 Egypt

**Keywords:** Mercury pollution, Artisanal gold mining, Nile River, Amalgamation, Exposure

## Abstract

The past decade witnessed the initiation and boom of the Artisanal and Small-scale Gold Mining (ASGM) activities in the hyper-arid southern Egypt. The ores are mined in the Eastern Desert and then transported to the densely populated farming communities in the Nile Valley, where the river provides the water resources needed for ore processing. In search for economic benefits, the poorly educated farmers with limited technical resources transformed their cultivated lands into ASGM operations, exposing themselves, their families, the residents, and the Nile ecosystems to several environmental and occupational health problems. Using integrated remote sensing, field, geochemical, and isotopic analyses, we report the first inventory of ASGM-related total mercury (THg) and methylmercury (MeHg) levels in tailings, amalgamation-tailing ponds, and surface and groundwater with emphasis on the Edfu city and its surroundings. The field and remote sensing-based mapping of ASGM activities reveals clustering around the Nile waterways and suggests interaction of Hg contamination sources with their surrounding receptors. Common ASGM practices include release of contaminated water from unlined amalgamation-tailing ponds into irrigation and drainage canals, and spreading of tailings over cultivated soils. In a short period (10 years), the released Hg contaminated multiple media, including the surface water, the shallow and deep aquifers, and possibly the soil, crops, and livestock. THg levels in amalgamation-tailing ponds (1200–8470 ng/L) are fourfold higher than US EPA and eightfold the WHO thresholds. The contaminated waters released from amalgamation-tailing ponds raised THg levels in surface water (irrigation canals: 50–100 ng/L; drainage canals: THg: > 200 ng/L) and groundwater (shallow and deep aquifers: 80–500 ng/L). Our findings highlight the need to extend the adopted approach to cover the entire length of the Nile River and its valley and the importance of conducting awareness campaigns to educate residents and health care providers about potential ASGM-related environmental and health hazards.

**Graphical Abstract:**

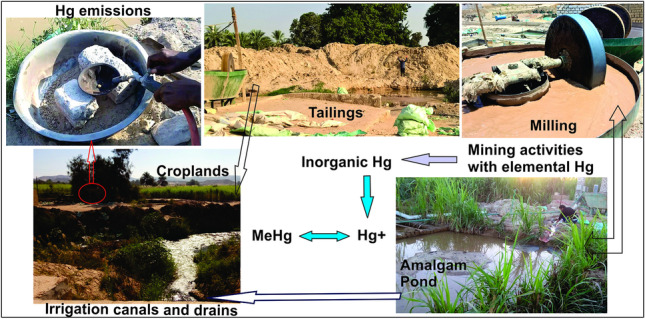

## Introduction

Artisanal and small-scale gold mining (ASGM) in developing countries is the largest source of global mercury contamination of surrounding ecosystems (Calao-Ramos et al.^2021^; Esdaile and Chalker 2018; Gerson et al. 2018; Gray et al. 2002; Mohamed et al. 2015; Niane et al. 2019; Steckling et al. 2017; Telmer and Veiga 2009; Veiga et al. 2009). There are two ways by which amalgamation in gold recovery is conducted. The first is by whole ore amalgamation (WOA), where mercury is in contact with 100% of the ore. This method releases large quantities of mercury from the polluted ASGM sites into the surrounding ecosystems (e.g., surface and groundwater). In the second method (concentrate amalgamation), the gold is concentrated before amalgamation using gold shaking tables. Mercury is then applied to the concentrate, and finally, gold is recovered by burning the gold (Au)-mercury (Hg) amalgam (Álvarez 2000; UNEP 2012).

The use of Hg in gold processing is considered illegal in most countries. Yet, amalgamation remains the preferred method by ASGM miners around the world, especially in Africa due to its availability, ease of use, and low cost (Balzino et al. 2015; Green et al. 2019; Hilson 2006; Hilson et al. 2007; Hilson and Vieira 2007; Limbong et al. 2003; Shandro et al. 2009; Veiga et al 2009). Approximately 380 to 450 tons of gold (t/Au) is produced annually from ASGM activities worldwide, with a gross income of $10.7 billion. The production is on the rise (Esdaile and Chalker 2018); for example, the ASGM activities produced 280 t/Au in Sudan from 2010 to 2015, which amounted to 85% of total gold produced in the country up to 2015 and reached 107 t/Au in 2018 (El Tohami 2018).

ASGM, a highly profitable activity, contributes to achieving several sustainable development goals (SDGs). The ASGM operations help end poverty and achieve food security and gender equality, where tens of millions of people in developing countries, both men (70%) and women (30%), live off their ASGM-raised incomes (Delve 2020). ASGM contributes to achieving economic growth goals, promotes peaceful and inclusive societies, and strengthens partnerships, given the cross-disciplinary nature of the ASGM processes. However, achieving these goals requires strict regulatory rules (Miserendino et al. 2013), a condition commonly absent in informal ASGM sites that are unlicensed and unregulated. However, ASGM activities can adversely affect the achievement of other SDGs, specifically, the clean water and the life underwater and on land goals. The ASGM sites release highly polluting materials (e.g., total mercury (THg) and methylmercury MeHg) and other carcinogenic metals associated with gold ores (WHO 2016) adversely affecting ecological and environmental sustainability.

Inorganic mercury from ASGM activities, the elemental Hg^0^, enters terrestrial and aquatic ecosystems and could be converted through methylation to a highly toxic organic form of methylmercury (MeHg: CH_3_Hg^+^) in soils, sediment, and water (Gerson et al. 2018). MeHg is more easily absorbed into the bloodstream via the gastrointestinal tract than elemental mercury due to its high lipid solubility (WHO 2016). Thus, it is among the hazardous waterborne contaminants in freshwater bodies. It is concentrated in aquatic biota and ingested by humans and fish-eating animals and therefore could pose a threat to human health and the ecosystems at large, even when present at low concentrations (Hong et al. 2012; Marvin-DiPasquale et al. 2008; Sams 2007; Weinhouse et al. 2021).

The direct exposure to elemental mercury vapors through inhalation, especially during the heating of the gold amalgam, affects the respiratory, neurological, and immune systems causing nausea, headache, fever, abdominal cramps, and diarrhea depending on the exposure duration and the concentration of inhaled mercury (WHO 2003). Exposure for a few hours to elemental mercury vapor (Hg^0^) with concentrations exceeding 1–2 mg/m^3^ can lead to acute bronchiolitis, pneumonitis, and pulmonary edema (Asano et al. 2000). In some cases, acute exposure can result in respiratory failure and death (Landrigan and Etzel 2013). Long-term exposure to lower levels of Hg^0^, which occurs in all ASGM sites, causes tremors and other neuropsychiatric symptoms such as fatigue, insomnia, depression, memory problems, and hypertension (Böse-O’Reilly et al. 2010). Methylmercury can penetrate the blood–brain barrier and accumulate in the central nervous system leading to a decrement in the intelligence quotient (IQ) in children (Liu et al. 2018) and neurologic diseases in adults. These include tingling in extremities, ataxia, muscle tremor, paralysis, and movement disorders (Gibb and O’Leary 2014) in addition to cardiovascular impairment (Roman et al. 2011), blindness, hearing impairment, and in some cases, death (WHO 2016).

Egypt — the most populous and arid of the Nile River riparian — is witnessing one of the highest water budget deficits worldwide, exceeding 40 BCM/year (Nikiel and Eltahir 2021). Currently, the Nile River represents the primary source of freshwater (i.e., more than 97%) for more than 100 million Egyptians, with a water share of 55.5 billion cubic meters per year (BCM/year) (El-Saadawy et al. 2020). The abovementioned water budget deficit will increase during the upstream filling and operation of the Grand Ethiopian Renaissance Dam (GERD), with a median water impoundment for all filling scenarios of ~ 9.5 BCM/year (Heggy et al. 2021). The expected additional water deficit encourages Egypt to explore solutions to mitigate potential shortages in irrigation water (Omran and Negm 2018) by reusing agricultural drainage water and maintaining the water quality in the waterways (Heggy et al. 2021). Unlike other countries worldwide, where ASGM operations have been conducted for decades in semi-arid to humid regions where freshwater resources are abundant (Delve 2020), the investigated ASGM operations were introduced in a hyper-arid environment. The hyper-arid conditions in Egypt and the scarcity of water resources in the Eastern Desert (Abdelmohsen et al. 2020), where gold ores naturally occur, forced miners to bring their ASGM operations to the Nile Valley in direct contact with the Nile River and its waterway derivatives.

Unfortunately, the farming communities at large in these areas perceive the hazard of exposure to THg and MeHg pollution as minimal to absent. This misunderstanding is partly related to the high levels of illiteracy in these communities (up to 25%; CAPMAS 2021). This situation is common in other countries as well. A questionnaire revealed that ASGM miners in Ghana are aware of the environmental impacts of mining activities on the water bodies and the environment. Yet, most miners believe that mercury is not harmful and does not enter the food chain (Gyamfi et al. 2022). In addition, the ASGM operations in the study area have been fairly recent in the past 10 years; thus, massive long-term health impacts have not yet been observed. The harmful impacts of the ASGM operations in the Nile Valley on the health of the miners are not limited to exposure to Hg. Gold ores in the Eastern Desert of Egypt are commonly associated with other highly carcinogenic metals. These include arsenic in hydrothermally altered rhyolite and Listvenite gold ores (Ramzey et al. 2021; Zoheir and Lehmann 2011) and lead in volcanogenic massive sulfide gold ores (Botros 2003). The release of those highly carcinogenic metals as secondary products during the ASGM operations compounds the above-reported health and environmental hazards. The inhalation and ingestion of these metals can lead to lung cancer, skin cancer, lead encephalopathy, and other serious health problems (WHO 2016). For example, the ASGM activities in Zamfara, Nigeria, in gold ores with high levels of lead resulted in a series of unexplained deaths of young children that have been later attributed to lead poisoning (Dooyema et al. 2012).

**Fig. 1:**
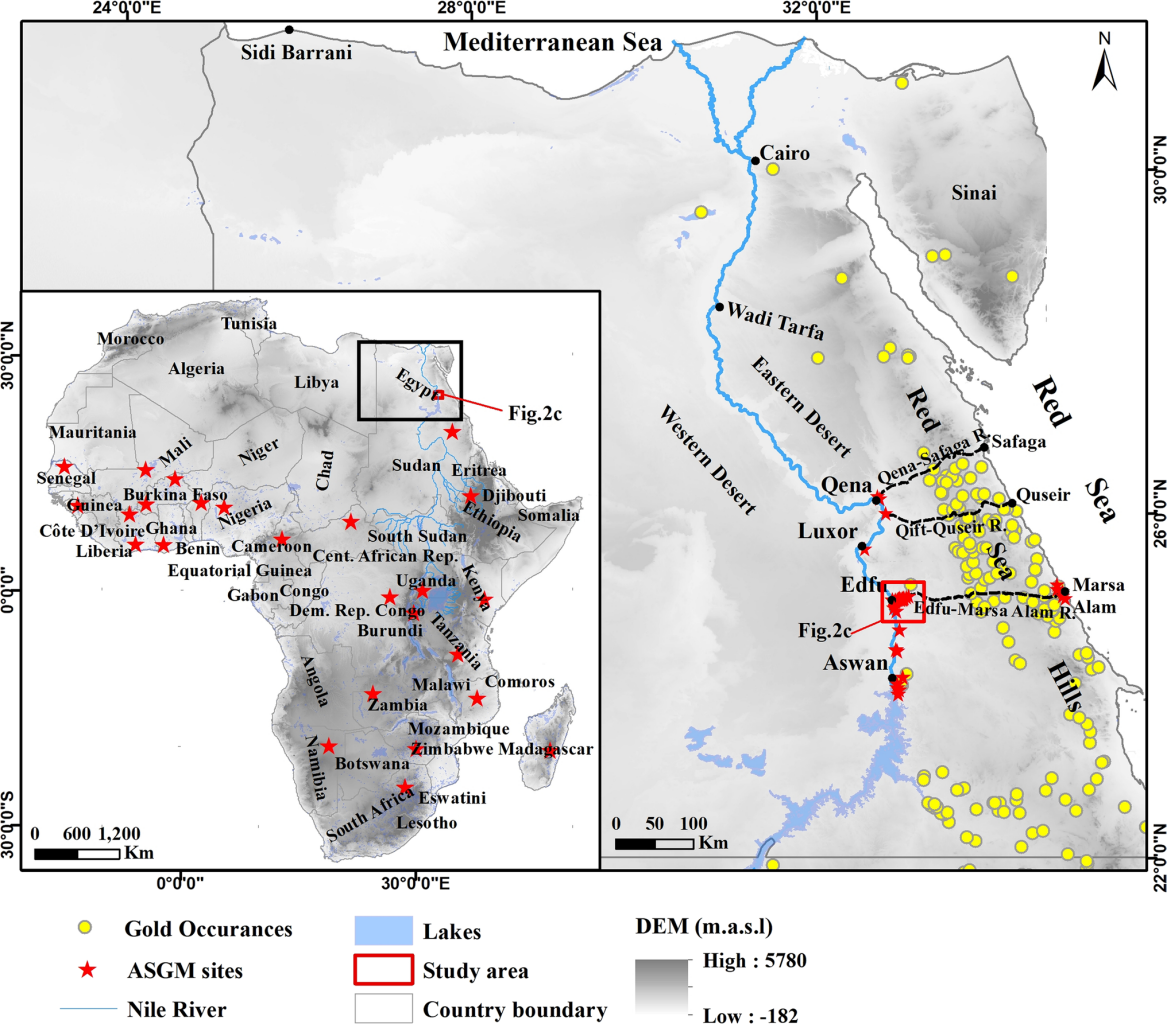
Locations of the ASGM operation sites along the Nile River and the Red Sea coastline, gold occurrences within the Red Sea Hills, where the ores are mined for the ASGM operations. The area covered by Fig. 2c is outlined by a red box. Inset is a compilation of Hg-contaminated sites (red stars) in Africa related to ASGM. Sources include: Benin (Armah 2013; Grätz 2009); Burkina Faso (Armah 2013; Jaques et al. 2008; Ouédraogo and Amyot 2013; Porgo and Gokyay 2016); Burundi (IPIS 2015; Machácek 2019); Cameron (Ralph et al. 2018); Central African Republic (IPIS 2019); Congo (Nkuba et al. 2019), Cote d’Ivoire (Mason et al. 2019); Egypt (this study); Ethiopia (Meaza et al. 2017); Ghana (Armah 2013; Basu et al. 2015; Donkor et al. 2006; Hilson 2002; Hilson et al. 2007; Rajaee et al. 2015); Guinea (Kolie et al. 2019); Kenya (Machácek 2019; Odumo et al. 2014); Madagascar (Cabeza et al. 2019); Mali (Armah 2013; MMSD 2001; SAICM 2010; Seccatore et al. 2014); Namibia (Podolský et al. 2015); Mozambique (Hilson et al. 2018; Mujere and Isidro 2015; Seccatore et al. 2014; Steckling et al. 2017); Nigeria (Awomeso et al. 2017; Samson et al. 2013; Uriah et al. 2013); Rwanda (Machácek 2019); Senegal (Armah 2013; Gerson et al. 2018; Niane et al. 2019); South Africa (Lusilao-Makiese et al. 2013; Oosthuizen et al. 2010); Sudan (El Tohami 2018; Mohamed et al. 2015); Tanzania (Nyanza et al. 2014); Uganda (Omara et al. 2019; Wanyana et al. 2020); Zambia (Kambani 2003; Podolský et al. 2015; Seccatore et al. 2014; Steckling et al. 2017); Zimbabwe (Mujere and Isidro 2015; Steckling et al. 2014; Veiga and Baker 2004)

**Fig. 2:**
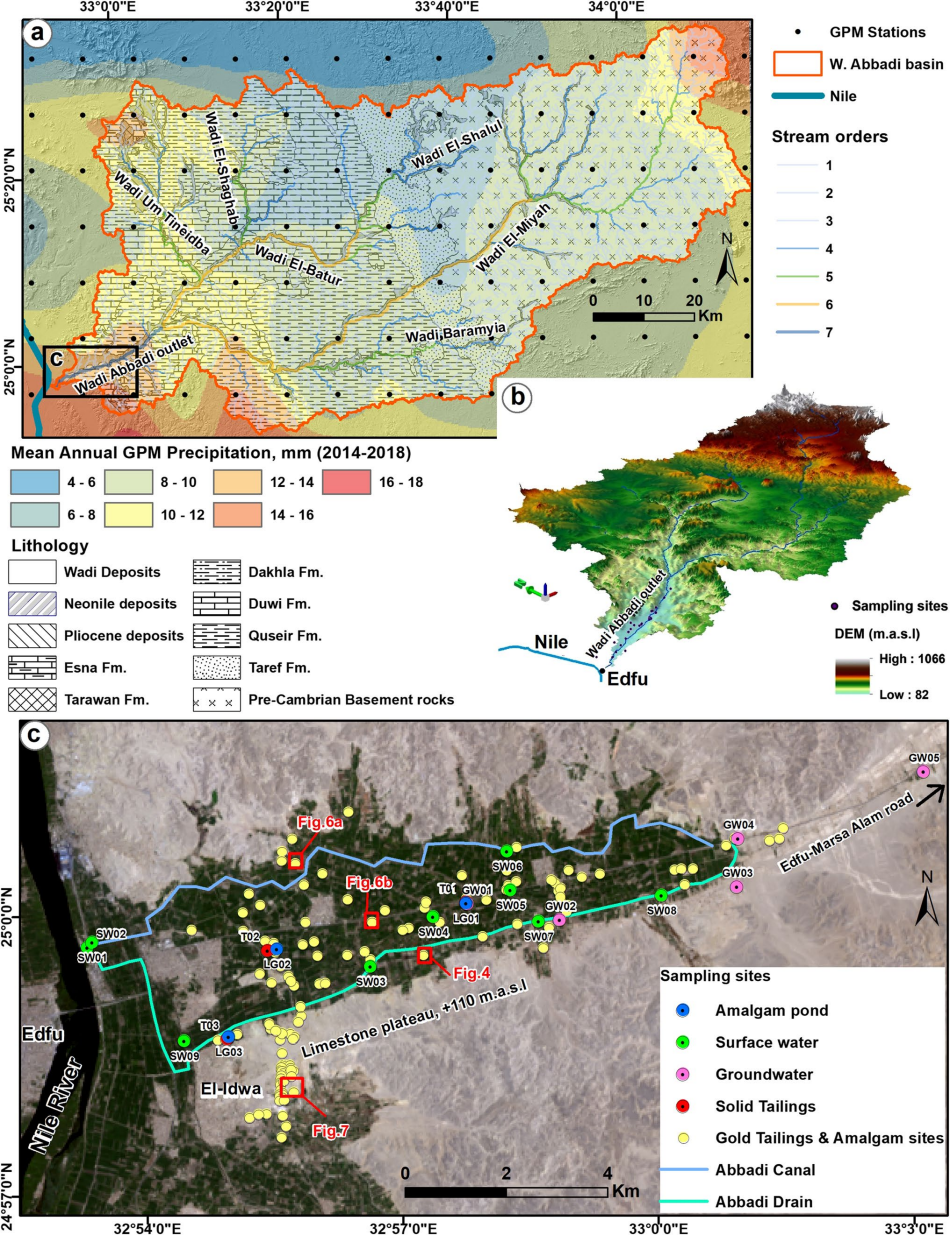
Location maps for the study area at the outlet of Wadi Abbadi and areas to the south (outlined by the red box in Fig. 1), Wadi Abbadi watershed, the irrigation and drainage canals, tailing and amalgamation sites, and sampling locations. **a**) Mean annual precipitation (2014–2018) over the Wadi Abbadi basin and its stream networks plotted over a simplified geological map for the basin (modified after Klitzch et al. 1987). The area covered by the black box in Fig. 2a is displayed in Fig. 2c. **b**) ArcScene-3D view of the Wadi Abbadi basin generated from ALOS PALSAR DEM (25-m spatial resolution and 12-m vertical exaggeration) shows Wadi Abbadi’s watershed, its source areas upstream, and its outlet near the Nile River. **c**) distribution of 130 gold tailings and amalgam sites and sampling sites of surface water (irrigation and drainage canals), groundwater, amalgamation-tailing ponds, and gold tailings. Also shown are the Abbadi irrigation and drainage canals. Note that the east–west trending Wadi Abbadi outlet is bound to the north and south by a limestone plateau that stands some 30 m above the wadi’s elevation (80 m.a.s.l.). Areas covered by Figs. 4, 6a, 6b, and 7 are outlined by the red boxes in Fig. 2c

The present study provides an assessment of THg and MeHg concentrations in different media within and surrounding the ASGM sites to address, at least in part, the apparent lack of awareness of the environmental implications of the newly introduced ASGM operations in the Nile Valley. We first define and apply specific criteria to map gold milling and amalgamation sites and monitor their evolution. We then report, for the first time, significant informal (unlicensed and unregulated) ASGM activities along Egypt’s Nile Valley (Fig. 1) and assess the environmental impacts of ASGM-related THg and MeHg contamination. The ASGM operations discharge contaminated waters into nearby irrigation and drainage canals and agricultural lands (Fig. 2). Specifically, this study aims at (1) mapping the distribution of milling operations and Au-Hg amalgamation sites within highly populated and cultivated areas, where residents of those areas are exposed to high levels of Hg contamination, as high as those experienced by the miners; (2) understanding the origin (natural or anthropogenic), type (elemental, organic), source (tailings, amalgamation-tailing ponds, and Hg emissions) of Hg contamination in surface and groundwater; (3) identifying the pathways by which Hg is transported from the sources (tailings, amalgamation-tailing ponds, and Hg emissions) to the receptors (surface and groundwater), and (4) assessing the adverse environmental impacts of the ASGM-related THg and MeHg contamination on surface and groundwater, and the socio-economic effects of these recent ASGM activities (~ 10 years) on the livelihood and sustenance of many of local residents. Our findings apply to ASGM activities in similar geological, hydrogeological, and/or operational settings along the Nile Valley (e.g., near Qift, Luxor, and Aswan cities; Fig. 1) and along the Red Sea coastline (e.g., Marsa Alam, Fig. 1) in Egypt, and elsewhere in Africa (Fig. 1 inset).

## Site description

Starting in 2011, ASGM operations in Egypt witnessed a progressive increase along the Nile Valley (e.g., Qena, Edfu, and Aswan), although the authorities banned such activities. The gold ores are mined from the Neoproterozoic volcano-sedimentary assemblages in the Red Sea Hills, yet they are not processed near the mining sites but instead transported to the Nile valley for processing (Fig. 1). The water availability and socio-economic aspects drive ASGM miners to transport the ores to the cities on the eastern bank of the Nile River, where water is available for gold processing, labor is cheap, and supply chains are proximal. However, the ASGM activities in highly populated cities along the Nile Valley, southern Egypt, could expose residents to high mercury contamination. Moreover, mercury contamination reaches different environmental media directly/or in a short time (e.g., surface, groundwater, soil, vegetation, fish, birds, and livestock).

Unfortunately, even first-order estimates of Hg used in ASGM gold operations in Egypt are challenging to obtain. Sales of gold produced from, and the equipment used in ASGM operations are informal (unlicensed and unregulated), unregistered, and not even available on global ASGM records (Delve 2020). Given such data’s absence, the ASGM activities’ environmental impacts in Upper Egypt remain ill-characterized and far from being understood. We start addressing those shortcomings by providing a detailed assessment of the nature, distribution, and environmental impacts of the ASGM activities in one affected city (Edfu). Future studies could potentially replicate our investigation in the study area over many ASGM sites within the Nile valley where the recent ASGM operations flourished.

The study area is proximal to and east of Edfu City (population: 460,232 in census 2017; source: www.capmas.gov.eg), which lies within the Nile Valley proper. The study area extends eastwards for some 10 km from the outlet of the east–west trending, Wadi Abbadi, where it discharges into the Nile River (Fig. 2a, b). Hereafter, this area is referred to as the Wadi Abbadi outlet. From east to west, the following rock units crop out within the Wadi Abbadi watershed (area: 6757 km^2^), Neoproterozoic basement Precambrian rocks, Taref, Quseir, Duwi, and Dakhla Formations (Upper Cretaceous), Tarawan and Esna Formations (Paleocene), Pliocene deposits, and Quaternary deposits (Hammad et al. 2015; Ibrahim et al. 2011; Klitzch et al. 1987) (Fig. 2a). The study area also encompasses additional ASGM sites located southwest of the Wadi Abbadi outlet and constructed over the limestone plateau, south of and parallel to Wadi Abbadi (El-Idwa village; Fig. 2c).

Farmers use the Abbadi irrigation canal to irrigate their lands east of the Nile and the Abbadi drainage canal to discard excess waters back to the Nile (Ibrahim et al. 2011; Fig. 2c). Precipitation is negligible (average annual rainfall 2014–2018: 4 to 18 mm; Fig. 2a) over the watershed, yet infrequent, heavy flash floods have been reported. For example, flash floods were reported on October 1991, November 1994, October 1997, and January 2010 (Ibrahim et al. 2011; Saber et al. 2020). Two main aquifers were reported from the study area: (1) a shallow unconfined to semi-confined aquifer, the Alluvial Aquifer (thickness: 20–300 m; lithology: alluvial deposits, gravel, sand, and silt); and (2) a deep aquifer, the Nubian Sandstone (NSS) Aquifer (lithology: sandstone of the Taref Formation, Fig. 2a); the aquifer is confined by the Quseir shale at a depth of 100–180 m and is the primary groundwater resource for Edfu and its surroundings (Hammad et al. 2015; Ibrahim et al. 2011; Mohammed et al. 2016; Sultan et al. 1997). Modern precipitation, irrigation return flow, and upward leakage from the NSS aquifer recharge the shallow alluvial aquifer (Hammad et al. 2015; Ibrahim et al. 2011; Mohammed et al. 2016), whereas precipitation during previous Pleistocene wet climatic periods recharged the deep aquifer (Sturchio et al. 2004; Sultan et al. 1997; Abotalib et al. 2016).

**Fig. 3:**
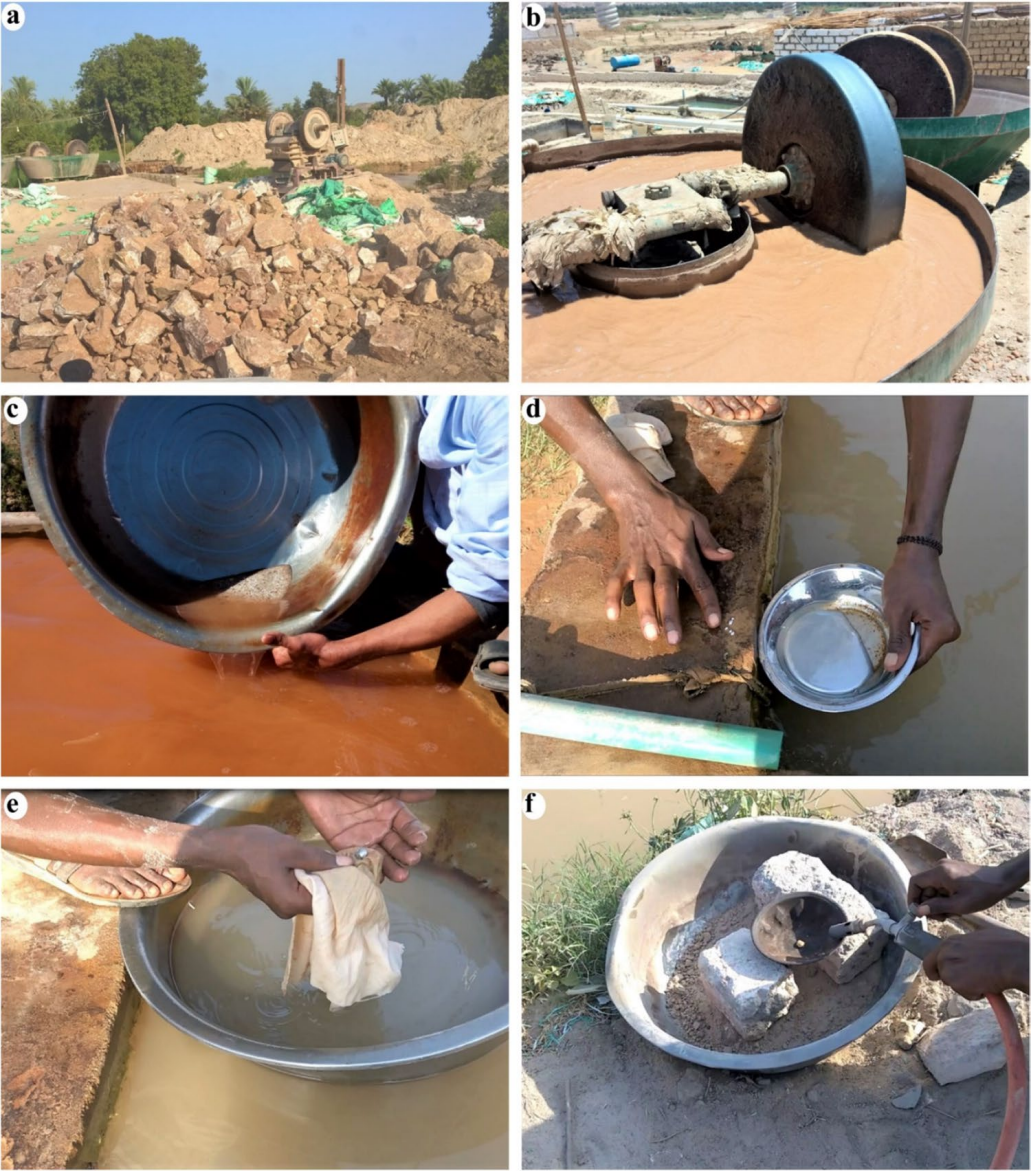
Field photographs show Au-Hg amalgamation and gold extraction processes associated with ASGM activities in the study area. **a**) Crushing ore rocks from small-scale and abandoned gold mines in the Eastern Desert. **b**) Milling crushed ore with elemental mercury in a wet pan to create a slurry. **c**) Washing out the slurry to separate the amalgamated residue. **d**) Hand-picking of the Au-Hg amalgam. **e**) Refining the amalgam by squeezing out excessive mercury using a cloth. **f**) Burning the amalgam to vaporize the mercury and produce gold sponge particles

The ASGM gold processing in the study area involves the following steps: (1) hauling the ore from abandoned gold mining sites in the central and southern sections of the Eastern Desert (Abu El-Leil et al. 2019) (Fig. 1), (2) crushing the ore to the appropriate size (Fig. 3a), (3) milling crushed ore using wet pan mills, where two iron discs rotate in a 1.5-m-wide pan (Fig. 3b); Hg is introduced to react with gold to form an amalgam and precipitate, and fine tailings are carried by overflowing water to amalgamation-tailing ponds where they settle, and water is recycled, (4) squeezing the Hg out of the gold amalgam using a cloth, and (5) torching the residue to produce gold sponge particles (Fig. 3c, d, e, and f). Hg vapors are released into the atmosphere, introduced into the environment, and possibly inhaled by the involved miners during the burning process. The use of Hg in gold extraction and the proximity of the ASGM operations to urban centers, agricultural lands, surface, and groundwater pose added risks not only to the local miners but to the population (> 5000; (www.capmas.gov.eg)) at large within the study area in two significant ways. Many of the villages located at or near the outlet of Wadi Abbadi do not have potable tap water; instead, they use surface and groundwater for drinking and daily activities. We show that the ASGM activities sites contaminate those two freshwater sources. Moreover, the ASGM activities in the study area produce large quantities of Hg-rich Au tailings (estimated 200–600 t/site each month) that eventually contaminate soil, vegetation, and livestock.

## Data and methods

We integrated field, geochemical, isotopic, and remote sensing datasets in the present study. The remote sensing data were used to map the distribution of lithologic units, tailings, amalgam sites, and irrigation and drainage canals. Geochemical analyses were conducted to measure THg and MeHg concentrations in solid tailings, amalgamation-tailing ponds, and surface and groundwater. We identified potential pathways for contaminant transport by delineating the areal extent of the Wadi Abbadi basin and its stream networks, estimating precipitation over the basin from satellite-based data, and conducting stable isotopic analyses (δ^18^O and δ^2^H) to investigate the source of the contaminated waters in the study area.

### Remote sensing and field investigations

**Fig. 4:**
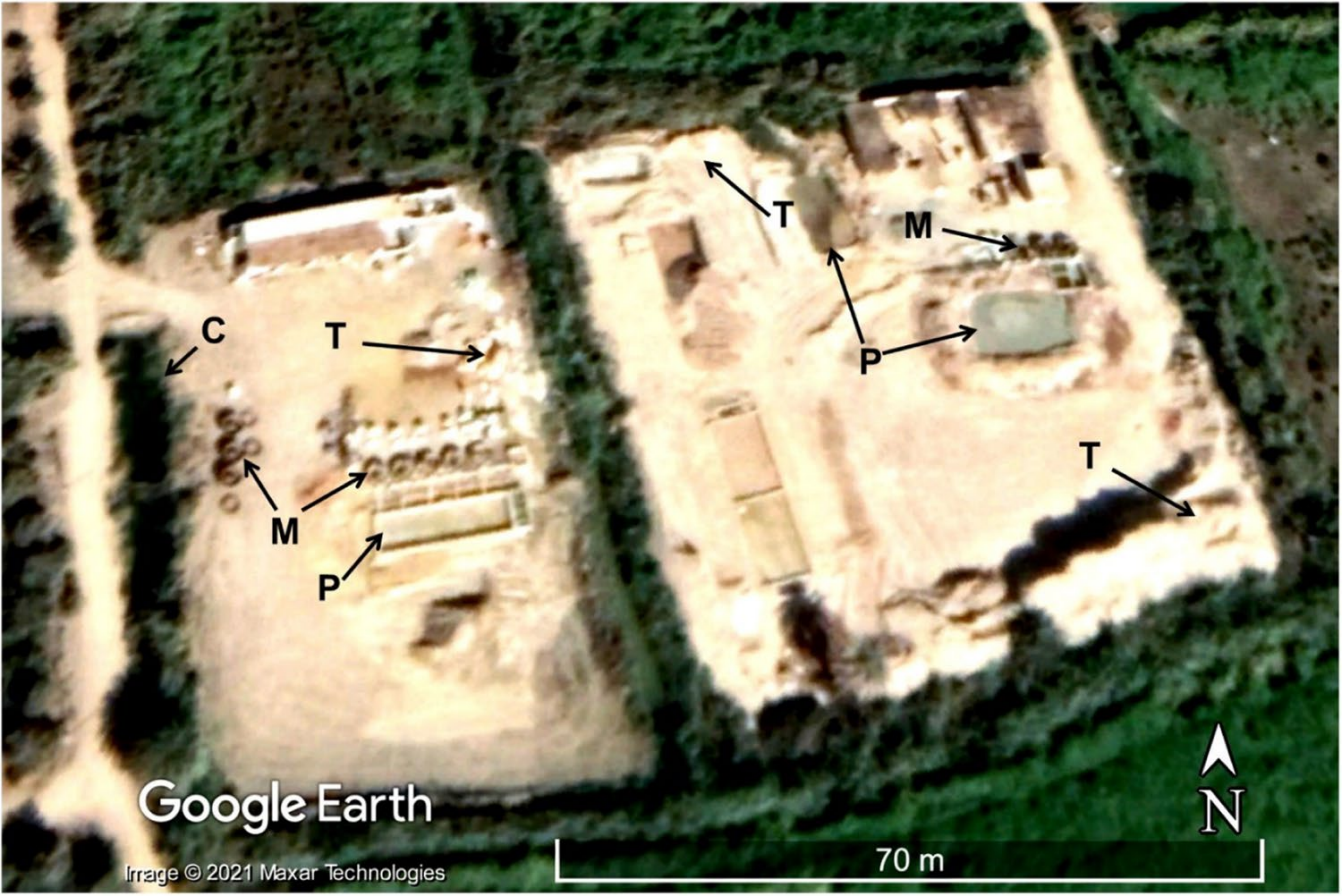
Use of geomorphologic and spectral characteristics of the gold tailings (T), amalgamation-tailing ponds (P), and wet-pan mills (M) displayed in a Google Earth high-resolution multispectral image to delineate gold processing sites in the study area. A red box in Fig. 2c outlines the area covered by Fig. 4

We extracted the distribution of lithological rock units within the study area from geological maps (Klitzch et al. 1987) (Fig. 2a), the watershed boundary and its stream networks (Fig. 2a) from ALOS PALSAR DEM (scenes: 3; spatial resolution: 25 m; source: Alaska Satellite Facility (ASF) website (https://vertex.daac.asf.alaska.edu/#)) using the Watershed/Hydrology module of Spatial Analyst Tools in ArcGIS 10.6 (Esri 2011), the daily precipitation over the watershed (2014–2018) from the Global Precipitation Measurement (GPM) data (Huffman et al. 2018) from the GPM website (https://pmm.nasa.gov/data-access/downloads/gpm) from which the average annual precipitation was calculated (Fig. 2a). The ALOS PALSAR DEM was also used to visualize the watershed in 3D view using ArcGIS 10.6 (Esri 2011). We used Google Earth high-resolution multispectral images (2016–2018) to map the distribution of gold tailings and amalgam sites within agricultural lands (Fig. 4), and land use maps to extract the irrigation and drainage canals (Fuller et al. 2012; Gao et al. 2018). Figure 4 shows the geomorphologic and spectral characteristics extracted from high-resolution Google Earth images, which allowed the identification of gold processing sites within the study area. We had to identify three components in a location to categorize it as a gold processing site: gold tailings (T), amalgamation-tailing ponds (P), and wet pan mills (M). The gold tailings appear as bright, closely spaced heaps or mounds of crushed ore that are conical or irregular in shape. The mounds are of limited lateral extent (< 10 m) and are a few meters high.

### Geochemical investigations

#### Sampling and analysis of mercury in tailings, surface water, and groundwater 

**Fig. 5:**
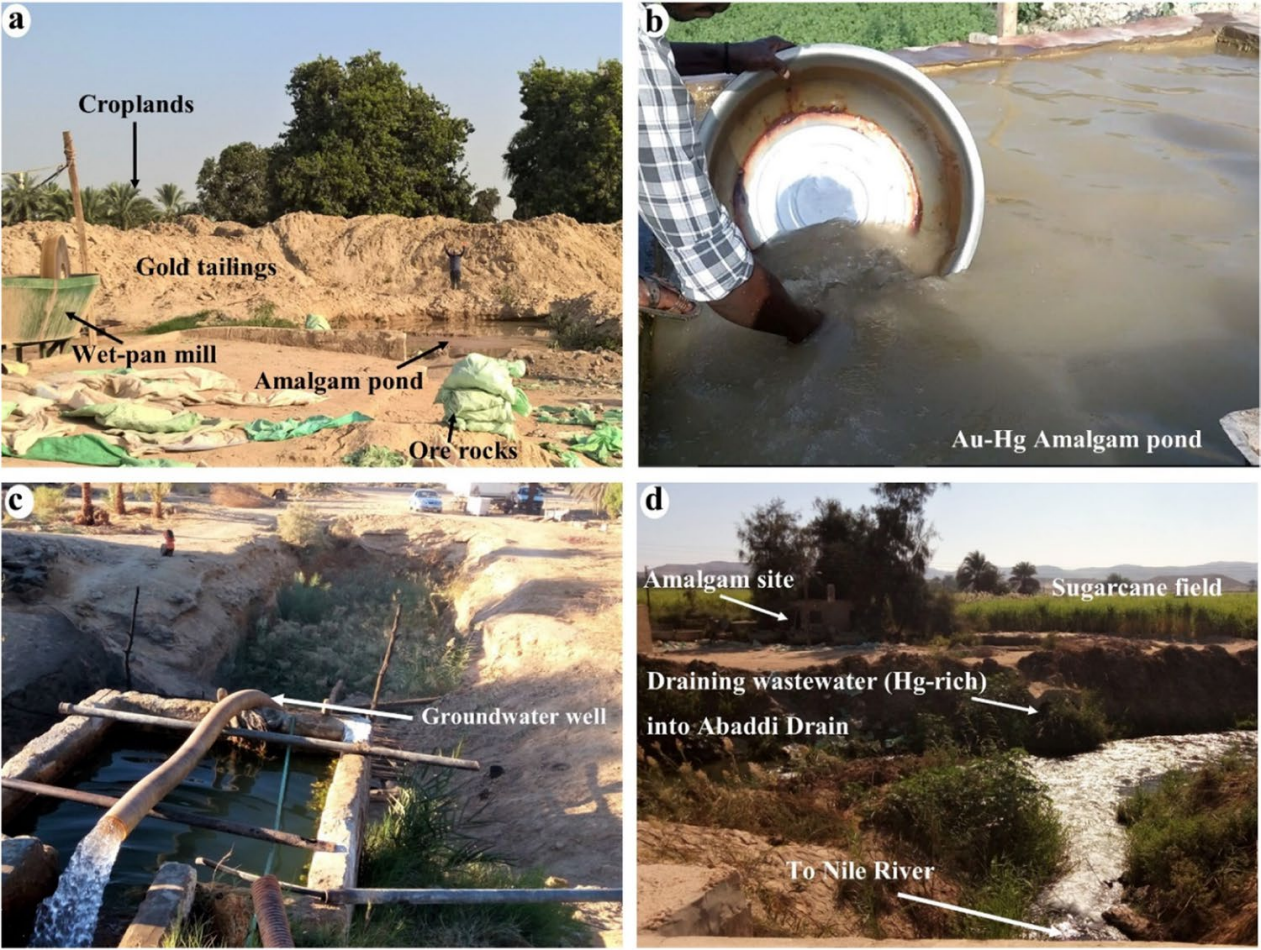
Field photographs show sample collection sites and settings (e.g., tailings, amalgamation pond, drainage canal, open wells) for THg and MeHg sample collection and analysis. **a**) Tailing sludge composite sample T02 produced from milling and amalgamation operations; **b**) surface water sample LG01 from an amalgam pond that overflows and channels Hg-rich wastewater into adjacent cultivated lands and sample T01 collected from a tailing pile within the same processing site; **c**) groundwater sample GW04 from an open well near a processing site; **d**) surface water sample SW02 from the Abbadi drainage canal, which collects wastewater from gold processing sites and channels it back to the Nile River

Composite samples of gold tailings (3 samples, Fig. 5a) from the gold processing sites or their immediate surroundings were collected in HDPE 500 g clean bags and frozen for mercury analysis (Marvin-DiPasquale et al. 2008). Surface water samples were collected (December 2018) from the Au-Hg amalgamation ponds (3 samples, Fig. 5b–c), irrigation and drainage canals (9 samples, Fig. 5e–f) within the gold processing sites or their immediate surroundings. Groundwater samples (5 samples) were collected from wells (Fig. 5d) near the gold tailings and Au-Hg amalgamation sites. The pH, Conductivity (EC), and Total Dissolved Salts (TDS) were measured in the field using Lovibond SensoDirect 150 Set. All field data and lab analyses are reported in Table 1. There is apparently no consensus in the protocols on whether THg and MeHg concentration should be reported for filtered or unfiltered water fractions (Bravo et al. 2018). We selected to collect unfiltered water samples as it better represents the risks associated with the consumption of unfiltered contaminated waters by the residents, livestock, and animals in our study area. Thus, the reported THg and MeHg concentrations in water samples in this study may reflect the contributions from suspended and dissolved Hg in the sample.

**Table 1:** Physico**-**chemical analysis of groundwater, surface water, and gold tailings near ASGM sites in the study area

	pH	TDS (mg/L)	EC (mS/cm)	δ^18^O (‰)	δ^2^H (‰)	THg (ng/L)	MeHg (ng/L)
*Groundwater*							
GW01	6.93	775.8	1.21	2.94	22.5	80	
GW02	7.01	1687	2.64	− 8.2	− 65.1	200	
GW03	7.03	3285	5.13	− 6.81	− 54.5	500	
GW04	6.95	9362	14.6	4.47	27.7	500	0.16
GW05	7.1	2495	3.9	− 8.33	− 63.4	80	
Mean	7.04	3520	5.5			272	
*Surface water (irrigation and drainage canals)*							
SW01	7.36	1053	1.65			50	
SW02	7.38	1069	1.67			210	
SW03	7.35	2643	4.13			200	0.064
SW04	7.01	4130	6.46			50	
SW05	6.97	2756	4.31			200	
SW06	7.2	5452	8.52			100	
SW07	6.34	2423	3.79			130	
SW08	6.52	2216	3.46			200	
SW09	6.87	2795	4.37			50	
Mean	7.03	2506	3.9			132	
*Au-Hg amalgamation-tailing ponds*							
LG01	6.55	2680	4.19			1200	0.56
LG02	7.01	2885	4.51			8470	
LG03	7.02	2373	3.71			1600	
Mean	6.86	2646	4.13			3756	
*Gold tailings*						(ng/g)	(ng/g)
T01						20,000	
T02						30,000	13.7
T03						10,000	
Mean						20,000	

The surface and groundwater samples (unfiltered) were collected in 250-mL polyethylene bottles, preserved by 1%v/v HNO_3,_ and tightly capped and stored below 4 °C (Eaton et al. 2005; Guédron et al. 2014; Niane et al. 2019). The Alluvial Aquifer was sampled from two hand-dug shallow wells: (1) GW01, where the aquifer is semi-confined by a silty clay layer at a depth of 10 m, and (2) GW04, where the aquifer is unconfined, and the depth to water table (DTW) is 1.5–3 m deep (Fig. 2c). The deep aquifer was sampled from two deep wells (GW03, GW05) and an artesian well (GW02).

We analyzed the samples of tailings, amalgamation-tailing ponds, and surface and groundwater for THg and MeHg concentrate ions in Maxxam Analytics Laboratory at Mississauga, Ontario, Canada, as reported in Table 1. THg concentrations in water samples were analyzed using cold vapor atomic fluorescence spectrometry following the laboratory and reference methods, CAM SOP-00453 and EPA 7470A m, respectively. MeHg concentrations in water and gold tailings samples were analyzed using a purge and trap-gas chromatograph-atomic absorption spectrophotometry analyzer and the US EPA 1631 method (US EPA 2002) for water, and the SW-846 Ch 3 2007 method for solid tailings (CCME 2016). The method detection limit (MDL) for THg in water samples is 0.2 ng/L per the Canadian guidance manual for environmental site characterization supporting human health risk assessment (CCME 2016). THg concentrations in gold tailing samples were analyzed using the cold vapor atomic fluorescence spectrometry following the lab and reference methods (CAM SOP-00453 and EPA 7471Bm, respectively). The MDL for THg in tailings samples is 5000 ng/g. Mercury concentrations in all procedure blanks were below the MDLs. Quality control for THg and MeHg analysis was maintained with field and method blanks, blank spikes, matrix spikes, certified reference materials, and sample triplicates. Recoveries on blank and matrix spikes were 84–93% in water samples and 80–87% in tailings samples.

#### Sampling for stable isotope analysis of groundwater

Groundwater samples (5 samples) were collected in tightly capped 50-mL polyethylene bottles. Stable isotope ratios of oxygen (^18^O/^16^O) and hydrogen (^2^H/^1^H) in water samples were measured at ISOTECH Laboratories in Champaign, IL, USA, using a Picarro cavity ring-down spectroscopy (CRDS) laser system (Lehmann et al. 2009). The isotopic compositions of groundwater samples are reported in Table 1 in the conventional delta (δ) notation (as δ^18^O and δ^2^H) in units of permil (‰) and with ± 0.1‰ and ± 0.5‰ analytical precision for oxygen and hydrogen isotope ratios, respectively.

## Results and discussion

### Mapping ASGM sites 

**Fig. 6:**
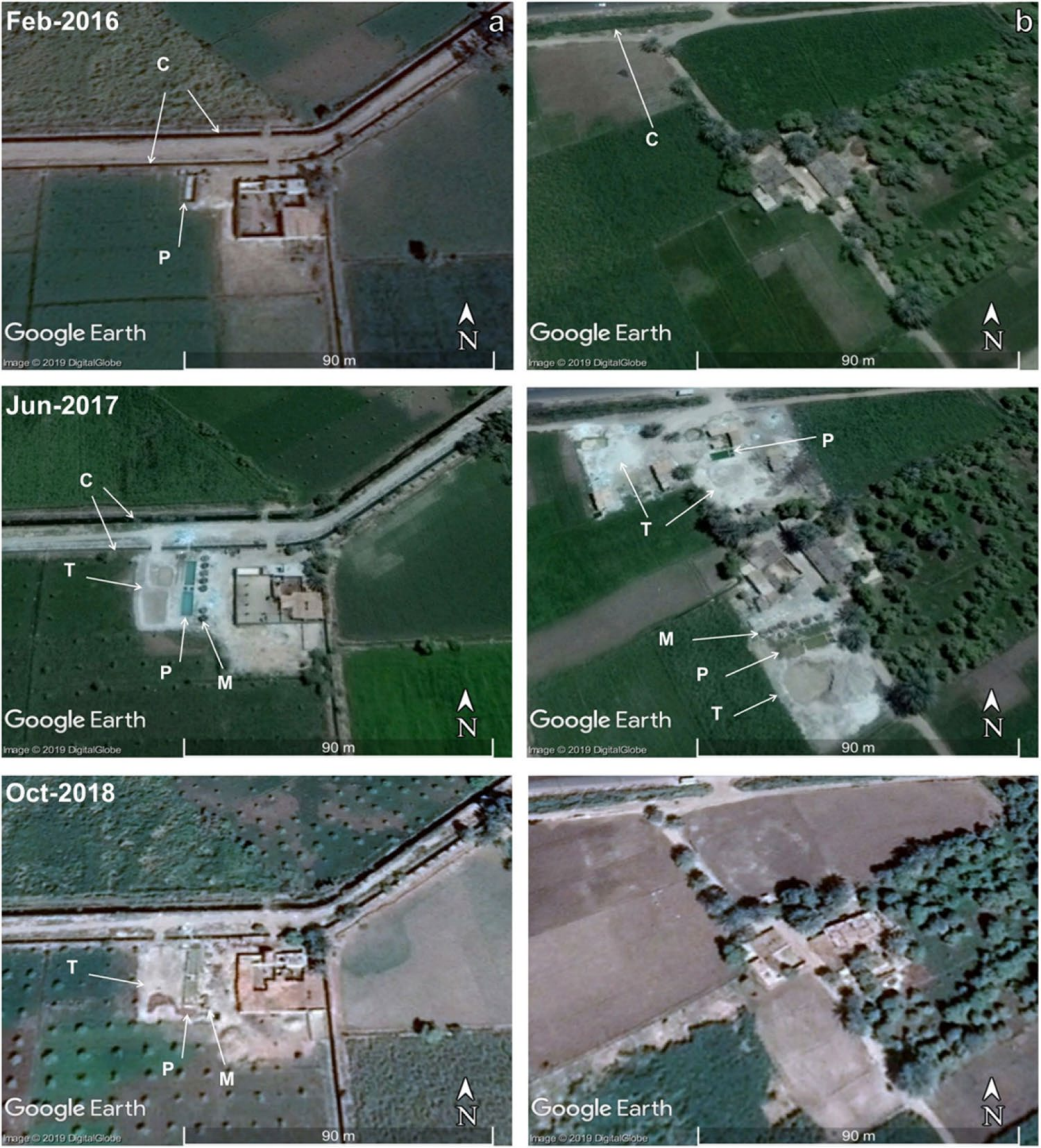
Google Earth high-resolution multispectral time-series images showing the distribution, setting, development (2016–2018), and potential impact of gold processing sites on the residents and ecosystems in two ASGM sites in the Wadi Abbadi outlet. **a**) Construction of a gold processing site proximal to an irrigation canal (C), where amalgam and slurry are washed, and the extracted Hg-rich waste is disposed of. Construction started in early 2016, and by late 2016 wet-pan mills were added, and gold processing operations were initiated. Operations continued in 2017 and 2018, as evidenced by the increase in tailing heaps (T) and amalgamation-tailing ponds (P). **b**) A short-lived site operational in 2017 was decommissioned by October 2018. Locations of sites a and b are outlined by red boxes in Fig. 2c

**Fig. 7:**
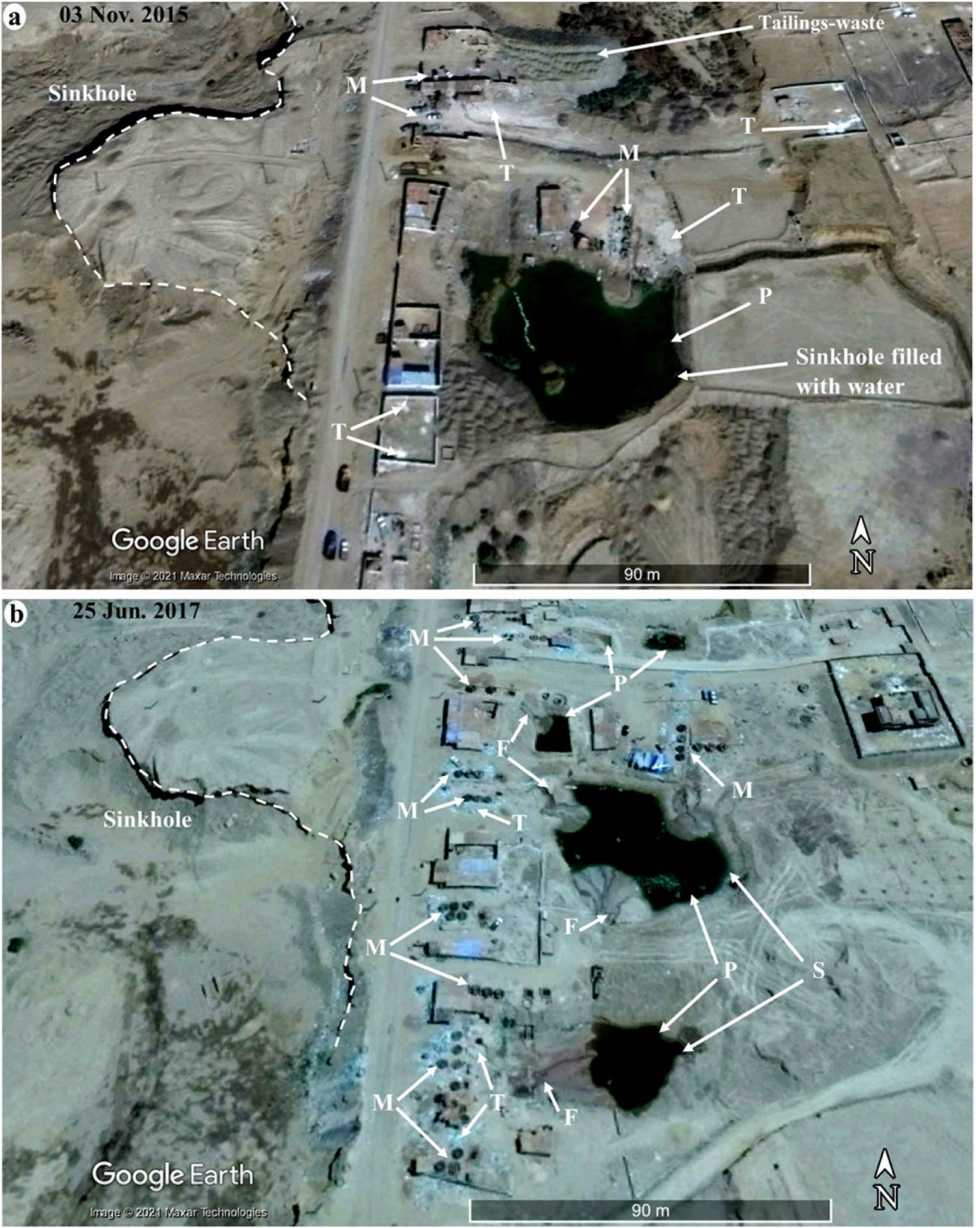
Temporal Google Earth high-resolution multispectral images showing the distribution, setting, development (2015–2017), and potential environmental impact of a gold processing site over the karstic plateau in El-Idwa village, southwest of Wadi Abbadi outlet, where depressions (outlined by white dashed lines) are being utilized as amalgamation ponds (P; dark polygons). Also shown is the distribution of associated wet-pan mills (M) and gold tailing heaps (T). Note the increase in gold processing activities between November 2015 and June 2017, as evidenced by the rise in the number of mills (M) and the fan-shaped drainage patterns (F) emanating from each of the ponds and flowing towards, and draining in, sinkholes (S) where the Hg-rich waste is disposed. A red box in Fig. 2c outlines the location of Fig. 7

Using the mapping criteria mentioned above, we identified over 130 active ASGM sites in the study area (Fig. 2c), ten of which were field verified. In those sites, tailings (total: 6000; average/site: 600), Hg wet pan mills (total: 62; average/site: 6 mills), and amalgamation-tailing ponds (total: 18; average/site: 2; Figs. 2c, 3, 4, 5a, 6, 7, and 8) were recorded. We mapped over 200 mills in El-Idwa village (Fig. 7) and some 600 mills in the Wadi Abbadi outlet (Fig. 6). We estimate some 28,000 t is produced in a month, given that each mill generates some 2 t of tailings/day (Esdaile and Chalker 2018; Veiga et al. 2009). The mills operate 6 days a week in the study area, two shifts/per day and eight h/shift. Moving the ASGM activities to areas proximal to the Nile River enabled year-round operation of the ASGM activities. The year-round operation increased the exposure time to mercury contamination and the release of mercury-contaminated water to the Nile River. We tracked the temporal variations in the number and size of the tailing heaps (T) and amalgamation-tailing ponds (P) in the Wadi Abbadi outlet. We found that in early 2016 tailings were transported, amalgamation-tailing ponds were constructed (Fig. 6a), and the sites were equipped with wet pan mills (M) shortly after. The production started at the end of 2016 and continued throughout 2017 and 2018. With few exceptions, most of the identified processing sites are still in place and are operational. One of those sites was operational up to 2017 but was decommissioned in 2018 (Fig. 6b).

**Fig. 8:**
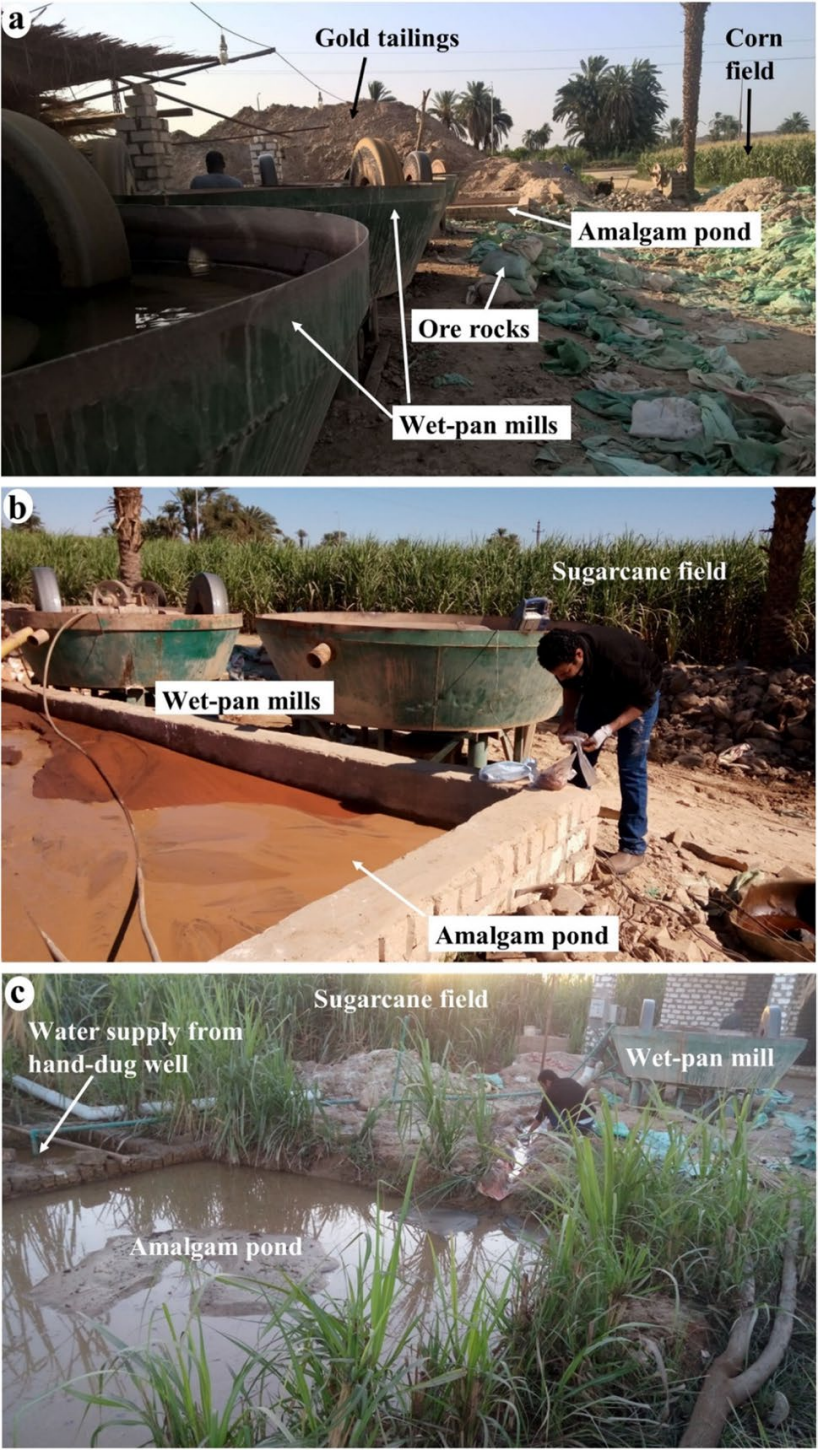
Field photographs show a typical setting for an ASGM site with its components (amalgamation-tailing ponds, wet pan mills, tailings) and its surroundings (croplands, canals, wells) in the study area. (**a**) Tailings and wet pan mills within croplands. (**b**) Amalgamation-tailing ponds and wet pan mills within croplands. (**c**) Amalgamation-tailing ponds within croplands. Figures demonstrate the direct contact between the sources of contamination (tailings and amalgamation-tailing ponds) and their Hg-rich wastewater that drains into nearby croplands and irrigation and drainage canals

**Fig. 9:**
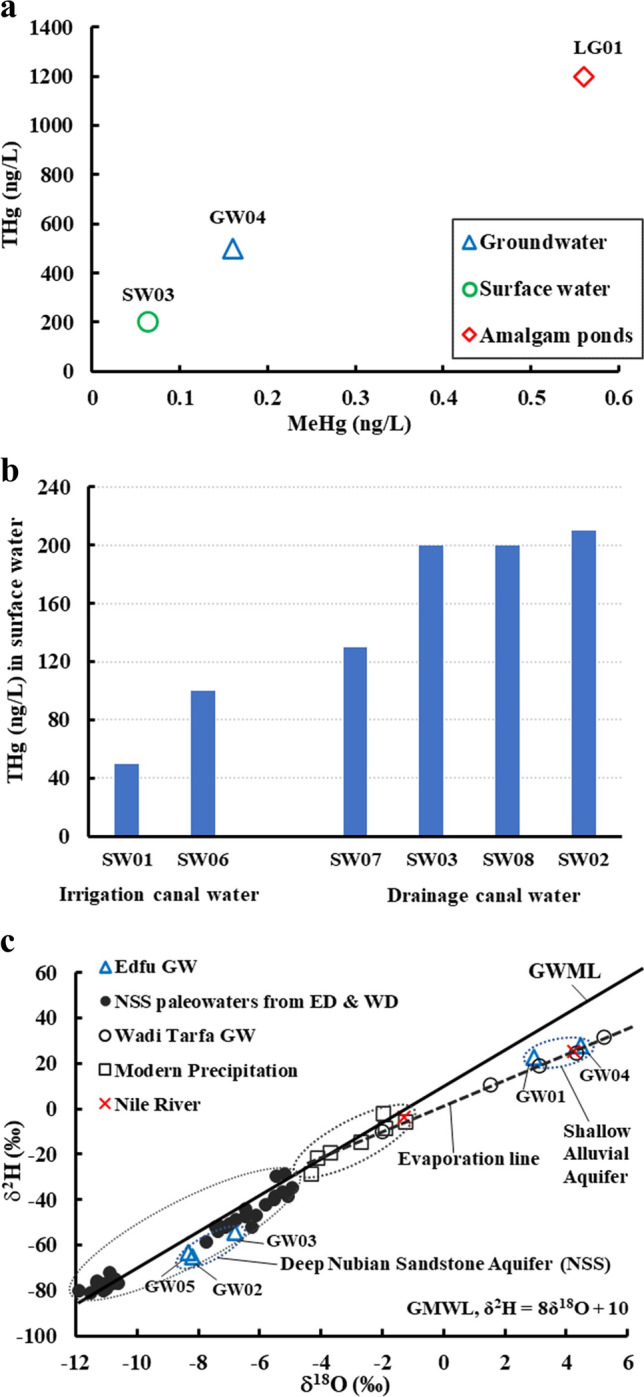
A) THg versus MeHg concentrations in groundwater, surface water, and amalgamation-tailing ponds (ng/L) in areas proximal to gold processing sites in the study area. **b**) Bar diagram for THg concentrations in surface water samples from the Abbadi Canal and Drain (Fig. 2c). **c**) δ^2^H versus δ^18^O isotopic compositions for groundwater from the shallow alluvial and the deep NSS aquifer. For comparison purposes, data are provided for the NSS aquifer paleowaters from the Eastern and Western Deserts (solid circles; Sultan et al. 2007, 1997), groundwater from Wadi Tarfa, Eastern Desert (open circles; Sultan et al. 2000), and modern rainwater from precipitation over Sidi Barrani and from the fractured basement aquifer that collects rainwater over the Red Sea Hills (open square; IAEA and WMO 1998; Sultan et al. 2007), and Nile River water (El Bakri et al. 1992; Sultan et al. 2000). Also shown is the global meteoric water line (GWML: δD = 8δ.^18^O + 10; (Craig 1961)

We recognized two different settings for the ASGM operations in the study area. The first and most common is within agricultural lands and proximal to the existing irrigation and drainage canals. The canals provide the water supply needed for washing the amalgam and slurry (Figs. 2c and 6). The amalgamation-tailing ponds are unlined, above-ground pools, square or rectangular in shape. They are typically 1 m high and meters to tens of meters on the side and filled with turbid waters, when operational. The walls are made from cemented bricks and the foundation from unlined permeable soil. The wet pan mills are dark rounded discs located proximal and subparallel to the amalgamation-tailing ponds. All remote sensing-based observations were successfully verified during our field trips in September and December of 2018 with an accuracy approaching 100%. The second setting is located over the higher grounds, namely the karstic limestone plateau, in El-Idwa village, southwest of Wadi Abbadi outlet (Figs. 2c and 7). The ASGM sites are located at large, proximal to natural depressions in this setting. The depressions resulted from the collapse of cavities within the karstic limestone giving rise to sinkholes (Abotalib et al. 2019; De Waele et al. 2011; Muzirafuti et al. 2020; Nawaz et al. 2020; Zhou and Beck 2008). The miners use pumps to raise the water from the lowlands (wadis) to the depressions on top of the adjacent plateau. They then utilize the depressions, now filled with water, as amalgamation-tailing ponds (Figs. 2c and 7b) and release the Hg-rich wastewater, a practice that is likely to contaminate the deep aquifer through infiltration and seepage through the karstic limestone substrate. In both settings, the tailings are not collected in tanks, but rather in heaps within the croplands and in direct contact with the soils. Extensive tailings, reaching up to 1000 t/site/month, are produced from gold milling operations and are stored at each site within the study area for long periods (up to 6 months) before being transferred outside the gold processing area (Figs. 3a, 6a, 7, 8, and 9a).

Many residents (individual families) in the Wadi Abbadi outlet run their milling operations in their house backyards (Figs. 6, 7, and 8). They are farmers by profession, many of whom are poorly educated, and they cultivate their lands with their family members. Following the introduction of the ASGM activities in the study area, and given the lucrative benefits of this industry, many farmers and their families got involved in the ASGM operations to secure additional resources while maintaining their farming activities. The proximity of the ASGM operations to the habitat of those farmer families and their collective participation in the gold processing operations poses direct occupational exposure to Hg contamination through vapor inhalation (Hong et al. 2012).

The exposure is exemplified by the lack of necessary resources to deal with mercury vapors and contaminated sediments. During our visits, we noticed that the mining operations lacked retorts or special fume hoods to avoid emitting Hg emission to the atmosphere. All workers had direct contact with mercury vapors and contaminated water and soils and did not use personal protective equipment (PPE) such as mercury or dust masks, boots, helmets, or gloves. Moreover, we saw a lack of awareness among the miners of the potential risks of mercury exposure (Fig. 3). The lack of PPE usage in the test sites contrasts with many other ASGM sites worldwide, providing training on PPE use. Examples include ASGM operations in Zimbabwe (Singo et al. 2022), Ghana (Ovadje et al. 2021), and Burkina Faso (Bugmann et al. 2022). PPE decreases mercury exposure and reduces its concentration in the human body. For example, the median urinary mercury concentration among workers in unregistered ASGM mines where PPE is not used is 18.5 μg/L, threefold higher than in registered mines where PPE usage is common (6.6 μg/L; Ovadje et al. 2021). The exposure could be indirect as well. If the Hg was largely in solution, the overflow from amalgamation-tailing amalgam ponds channeled into nearby croplands and irrigation and drainage canals can potentially contaminate vegetation, crops, soil, birds, and livestock.

### Mercury and methylmercury in tailings and amalgamation-tailing ponds

Field observations with those interpreted from remote sensing datasets suggest a direct Hg contamination within a source-receptor cycle. In this cycle, fine-grained tailings and amalgamation-tailing ponds (Hg-rich) are located within or near croplands (e.g., corn and sugarcane fields; Fig. 8), tailings are mixed with or spread over the cultivated land, and contaminated waters from the ponds are released into the nearest irrigation or drainage canals. The canals return contaminated waters to the Nile River. Moreover, the ponds lack liners, and their contaminated waters leak, overflow, or spill into the adjacent croplands (Figs. 2c, 5, 6, 7, 8, and 9a). The fine-grained Hg-rich tailings that accumulate within the ponds are offloaded and stored near the pond. The stored tailings are mixed with soils in the nearby croplands, exposing soils to contamination (Fig. 8a–c). The abovementioned activities and settings are likely to cause THg and MeHg contamination in nearby receptors (e.g., soil, croplands, vegetation, surface and groundwater, birds, and livestock) (Figs. 5 and 8a–c) and pose health risks to local miners and residents.

Samples from the tailings (T01–3) in the study area have relatively high levels of THg (10,000–30,000 ng/g) (Table 1). The THg levels in the tailings are within the reported range (20–54,600 ng/g) of ASGM sites in Senegal, Chile, and Ghana (Gerson et al. 2018; Leiva and Morales 2013; Niane et al. 2019; Rajaee et al. 2015). The MeHg concentration in the investigated tailings (13.7 ng/g) and the percentage of MeHg as a function of THg (%MeHg: 0.05%) are at the low end of the range (2.3–75 ng/g; 0.03–4.4%; Table 2) reported in Senegal, Ghana, and Suriname (Gerson et al. 2018; Gray et al. 2002; Niane et al. 2019). One explanation for the presence of MeHg in the tailings in the study area is the existence of conditions promoting methylation (Bigham et al. 2017) including: (1) high sulfur and iron content in the gold ores in Egypt (Ramzey et al. 2021; Zoheir and Lehmann 2011; Botros 2003), (2) high air surface temperature throughout the year, where the average annual air temperature is 26.8 °C, and reaching 42.2 °C in June (Galal et al. 2020), (3) mixing of tailings with soils rich in organic content from the surrounding croplands, and (4) tailings are subjected to cycles of wetting and drying. The wet tailings from the amalgamation-tailing ponds are transferred to the nearest tailing heap, where they dry under the hyper-arid conditions in the study area. Given the presence of all of the above-mentioned site characteristics that promote methylation, we expect that methylation and bioavailability of THg in the study area to increase with time (De Bakker et al. 2021; Guimarães et al. 1995).

**Table 2:** Total mercury (THg) and methylmercury (MeHg) concentrations in groundwater, surface water (irrigation and drainage canals), Au-Hg amalgamation-tailing ponds, and gold tailings from locations proximal to gold processing sites in the study area. For comparison purposes, THg and MeHg concentrations from ASGM sites in Africa and other parts of the world are provided, in addition to international contaminant levels for THg and MeHg

Sample type	THg	MeHg	Location	References
*Gold tailings (ng/g)*				
	5.5–200	< 0.02–1.4	ASGM, Suriname, South America	(Gray et al. 2002)
	77–2917	5–75	ASGM, Ghana	(Donkor et al. 2006)
	50–290		Techatticup Gold Mine, USA	(Sims and Francis 2008)
	100–27,610		ASGM, Indonesia	(Hidayati et al. 2010)
	16–4230		ASGM, West coast of India	(Ramasamy et al. 2012)
	13,600		ASGM, Andacollo, Chile	(Leiva and Morales 2013)
	15–21		ASGM, Nigeria	(Samson et al. 2013)
	2580	10	ASGM, Gauteng, South Africa	(Lusilao-Makiese et al. 2013)
	5.8–1759		ASGM, Tanzania	(Nyanza et al. 2014)
	140–8900		ASGM, Kenya	(Odumo et al. 2014)
	196–1187		ASGM, Colombia	(Pinedo-Hernández et al. 2015)
	3770–54,600		ASGM, Ghana	(Rajaee et al. 2015)
		5.2	Gold mines, North Carolina, USA	(Singer et al. 2016)
	7500	4.2	ASGM, Senegal	(Gerson et al. 2018)
	20–2400	2.3–8	ASGM, Senegal	(Niane et al. 2019)
	38–726	0.21	ASGM, Cote d’Ivoire	(Mason et al. 2019)
	10,000–30,000	13.7	Edfu, Egypt	*This study*
*Au-Hg amalgamation-tailing ponds (ng/L)*				
	1–930	0.05–3.8	ASGM, Suriname, South America	(Gray et al. 2002)
	1.5–8230		ASGM, Indonesia	(Hidayati et al. 2010)
	117–973	2–10	ASGM, Senegal	(Niane et al. 2019)
	1200–8470	0.56	Edfu, Egypt	*This study*
*Surface water (ng/L)*				
	25–40	0.03–0.56	ASGM, Guiana, South America	(Boudou et al. 2005)
	28.7–420	< 0.028–19.64	ASGM, Ghana	(Donkor et al. 2006)
	0.5–2030		ASGM, Indonesia	(Hidayati et al. 2010)
	14–25		ASGM, Nigeria	(Samson et al. 2013)
	220	0.04–2.12	ASGM, Gauteng, South Africa	(Lusilao-Makiese et al. 2013)
	400–21,380	0.01–0.21	ASGM, Burkina Faso	(Ouédraogo and Amyot 2013)
	< 1000–47,800		ASGM, Tanzania	(Nyanza et al. 2014)
	22	0.037	ASGM, Senegal	(Gerson et al. 2018)
	5.8–86.3	0.2–6.9	ASGM, Senegal	(Niane et al. 2019)
	6.6–53	0.11	ASGM, Cote d’Ivoire	(Mason et al. 2019)
	50–210	0.064	Edfu, Egypt	*This study*
*Groundwater (ng/L)*				
	14		ASGM, Indonesia	(Hidayati et al. 2010)
	223		ASGM, Gauteng, South Africa	(Lusilao-Makiese et al. 2013)
	80–500	0.16	Edfu, Egypt	*This study*
*International contaminant levels of THg and MeHg in ground-and surface water (ng/L)*				
	2000			MCL (US EPA 2018)
	1000			(WHO 2018)
	26	4		(CCME 2007)
	1000			(EU Directive 98/83/EC 1998)

Water samples from the Au-Hg amalgamation-tailing ponds (LG01–03) show high levels of THg (1200–8470 ng/L) (Table 1 and Fig. 9) and are comparable to those reported in Africa and worldwide (Gray et al. 2002; Hidayati et al. 2010; Niane et al. 2019) (Table 2). For example, the THg levels in the ponds in the study area are higher than those reported in ASGM amalgamation-tailing ponds in Senegal, West Africa (117–973 ng/L; Table 2; (Niane et al. 2019)). The THg levels (1200–8470 ng/L) in the amalgamation-tailing ponds exceed by as much as four times the Hg maximum contaminant level (MCL; 2000 ng/L) for aquatic life (US EPA 2018) and are eight times higher than the Hg standard (1000 ng/L) recommended by both WHO (WHO 2018), and the European Union standard for drinking water (EU Directive 98/83/EC, 1998) (Table 2). The ponds are not covered and are accessible to birds and livestock, a setting that magnifies the adverse impacts of those ponds (Figs. 5b and 8c).

The MeHg concentration in the amalgamation-tailing ponds (0.56 ng/L, 0.05%) is low compared to levels reported from amalgamation-tailing ponds in Africa, such as those from Suriname (0.05–3.8 ng/L; 0.3–8.5%; Table 2; (Gray et al. 2002)) and Senegal (2–10 ng/L; 0.3–8.5%; Table 2; (Niane et al. 2019)). The MeHg concentration in the amalgamation-tailing ponds in the study area is lower than the long-term guideline (4 ng/L) for protecting aquatic life set by the Canadian water quality in freshwater (CCME 2007). The low %MeHg in the amalgamation-tailing ponds is probably due to the short operational period of the ASGM activities in the study area and the oxygenation associated with the continuous mixing of the pond and irrigation waters, which could impede the methylation process (Chortek 2017; Mailman et al. 2006).

### Mercury and methylmercury in surface water 

The THg levels (50–210 ng/L; Table 1) in the investigated surface water samples are lower than the Hg MCLs of US EPA (2000 ng/L) and the WHO drinking water standards (1000 ng/L; Table 2) yet exceed the US EPA standards (12 ng/L) for protection against chronic Hg effects on aquatic life (US EPA 1992). The THg levels are high compared to contaminated rivers elsewhere. Examples include Gambia and Bantako rivers in Senegal (Gerson et al. 2018; Niane et al. 2019), the Manyara River in Nigeria (Samson et al. 2013), and the Sinnamary River in Guiana (10–80 ng/L; (Boudou et al. 2005)). Natural freshwater generally contains < 5 ng/L THg and 0.02 to 0.1 ng/L MeHg (Ullrich et al. 2001).

The MeHg concentration (0.064 ng/L, 0.03%) in surface water samples in this study (Fig. 8a) is below the Canadian water quality guideline (4 ng/L) for protecting aquatic life (CCME 2007) (Table 2). However, it exceeds those reported for the Bantako River in Senegal (0.037 ng/L; (Gerson et al. 2018)) and is within the range (0.01–19.64 ng/L) of the Hg-contaminated rivers in Burkina Faso, South Africa, and Ghana (Donkor et al. 2006; Lusilao-Makiese et al. 2013; Ouédraogo and Amyot 2013) (Table 2). The %MeHg in the surface water in this study (0.03%) is low compared to the 1.5% reported from the Idrija River in Slovenia (Hines et al. 2000) and the range (0.3–8.5%) in the Gambia River in Senegal (Niane et al. 2019). The ASGM operations have impacted the Idrija River for the past 500 years and the Gambia River throughout the past two decades. The more recent ASGM activities in the study area over the past 10 years could explain the relatively low %MeHg values in the area.

The Abbadi irrigation canal channels Nile waters to the farmlands in the east, and the Abbadi drain carries excess water back to the Nile River. Samples from the irrigation canal (e.g., SW01 and SW06) have moderate THg levels (THg: 50–100 ng/L) compared to those from the drainage canals that have relatively high THg levels (SW02, SW03, and SW08; THg: > 200 ng/L; Fig. 9b). These emerging levels of THg in the drain water, if left unmanaged, could threaten the Nile River ecosystem in the near future and merit early intervention by the local authorities to take necessary precautionary measures.

### Mercury and methylmercury in groundwater 

In order to understand the correlation between surface processes and groundwater in the study area, it is necessary to determine the recharge sources to the aquifers. Water’s hydrogen and oxygen isotope ratios are excellent indicators of the origin and evolution of groundwater in general and in Egypt in particular (Clark and Fritz 1997; Sultan et al. 2008; Abotalib et al. 2021). The sampled groundwater for δ^18^O and δ^2^H isotopic compositions in the study area came from two aquifers: (1) samples (GW01 and -04) from the shallow Alluvial Aquifer flooring Wadi Abbadi and (2) samples (GW02, -03, and -05) from the deep NSS aquifer. Inspection of Fig. 9c shows that the isotopic compositions of the deep and shallow aquifers differ. The alluvial aquifer samples have enriched isotopic compositions (δ^18^O: 2.94 to 4.47‰ and δ^2^H: 22.5 to 27.7‰; Table 1, Figs. 2c and 9c) similar to those reported from the alluvial aquifer flooring of Wadi Tarfa (Sultan et al. 2000) with a recharge origin of either evaporated modern precipitation or through a hydraulic connection with the Nile Valley aquifer (RIGW 1994, 1988). On the other hand, groundwater samples from deep wells have depleted compositions (δ^18^O: − 8.33 to − 6.81‰ and δ^2^H; − 65.1 to − 54.5‰; Table 1 and Fig. 9c) similar to those reported from the NSS aquifer in the Eastern and Western Deserts (Sultan et al. 2007, 1997).

In general, groundwater samples from the shallow and deep aquifers have THg concentrations (80–500 ng/L; Table 1) lower than the Hg MCLs of the US EPA (2000 ng/L) and the WHO (1000 ng/L) (US EPA 2018; WHO 2018) (Table 2). Similarly, the MeHg (0.16 ng/L, 0.03%; Table 1) in groundwater is lower than the Canadian water quality guideline (4 ng/L) for the protection of freshwater aquatic life (CCME 2007) (Table 2). However, the THg levels in surface water (50–210 ng/L) and groundwater (80–500 ng/L) exceed by as much as 17 and 40 times, respectively, the standard (12 ng/L) recommended by the US EPA for the protection against chronic Hg effects to aquatic life (US EPA 1992). Given that the amalgamation-tailing ponds in the study area are either unlined above-ground pools (i.e., not tanks) or sinkholes excavated in highly fractured carbonates, the ASGM sites located within the study area and those that occur east of and upstream from the study area could be primary sources of Hg contamination in the groundwater (Fig. 2c). In particular, areas where mining activities occur over the karstic plateau (Fig. 7), the leakage from the unlined amalgamation-tailing ponds takes place through the karst system where the contaminants are rapidly transported downwards through preferred pathways (networks of faults, fractures, karst topography, and sinkholes) into the underlying NSS aquifer with minimal filtration due to the high vertical hydraulic conductivity along the preferred pathways (Abotalib et al. 2019; Sultan et al. 2007). A similar model for rapid groundwater flow through networks of faults and karst and recharge of the Eastern and Western Desert aquifers was validated using integrated geophysical, isotopic, and hydrological modeling approaches (Abdelmohsen et al. 2020, 2019; Hussien et al. 2017; Yousif et al. 2020). Deep fossil aquifers have been long assumed to be immune to contamination. However, recent studies have shown that 50% of these fossil aquifers receive significant contributions from modern water (Jasechko et al. 2017). Our findings highlight the risks associated with carrying out the ASGM practices over the karstic plateau in El-Idwa village, especially the use of natural depressions (fast flowing karst system) as amalgamation-tailing ponds (Fig. 7).

## Summary and implications

Using integrated remote sensing, field, geochemical and isotopic analyses, we assessed the distribution and environmental impacts of the recently developed (past decade) ASGM activities over Edfu city and its surroundings in south Egypt. The field and remote sensing-based mapping of ASGM activities revealed clustering around the Nile waterways where over 130 active gold tailings and Au-Hg amalgamation sites were mapped. We report the first inventory of ASGM-related THg and MeHg levels in tailings, amalgamation-tailing ponds, and surface and groundwater. The THg concentrations in tailings are 10,000‒30,000 ng/g, in amalgamation-tailing ponds, 1200‒8470 ng/L, in surface water, 50‒210 ng/L, and in shallow alluvial and deeper NSS aquifers, 80‒500 ng/L. The concentrations of MeHg in tailings are 13.7 ng/g (0.05%), in ponds, 0.56 ng/L (0.05%), in surface water, 0.064 ng/L (0.03%), and in groundwater, 0.16 ng/L (0.03%).

The hydrogeological investigation suggest that amalgamation-tailing ponds represent the major source of contamination in the study area. The ponds are either unlined, above-ground pools structures with permeable soil foundation in the lowlands, or sinkholes excavated in highly fractured carbonates over the plateau. They discharge into and contaminate nearby, croplands and irrigation and drainage canals, where the THg concentration could reach up to 210 ng/L, exceeding by as much as 17 times the standard (12 ng/L) recommended by the US EPA for protection against chronic Hg effects on aquatic life. Our investigations suggest direct contact between the reported high Hg sources (e.g., tailings and amalgamation-tailing ponds) and receptors (soil, croplands, surface-and groundwater, birds, livestock, local miners, and residents). This setting, if left unmanaged, would pose serious threats to the population and ecosystems in the near future, where the reported Hg levels are expected to accelerate with expanding the ASGM activities.

The MeHg concentrations in tailings are 13.7 ng/g (0.05%), in ponds 0.56 ng/L (0.05%), in surface water 0.064 ng/L (0.03%), and in groundwater 0.16 ng/L (0.03%). The ASGM-related THg levels are also reported from the shallow alluvial and deeper NSS aquifers (THg: 80–500 ng/L; MeHg: 0.16 ng/L, 0.03%). The MeHg concentrations in pond, surface, and groundwater are below the Canadian water quality guideline (4 ng/L) for the protection of aquatic life (CCME 2007), yet it is worthy to report that the rapidly expanding ASGM activities in south Egypt could result in acceleration of MeHg concentration to threatening levels.

We recommend initiating environmental health awareness campaigns targeting poorly educated miners, their families, residents, and local health care providers. The campaigns should address the ASGM-related environmental and health hazards and the importance of PPE in minimizing exposure to THg, MeHg, and other associated carcinogenic metals.

The WOA mining operation remains the preferred ASGM method in Egypt, NE Africa, Asia, and South America even though some 70% of Hg is lost and gold recoveries do not exceed 30%. Replacing ASGM with modern eco-friendly amalgamation methods is a costly investment that requires elaborate tailored processes for varying ore types and mining sites (Niane et al. 2019; Veiga et al. 2009). Efforts should be directed towards promoting and regulating alternative eco-friendly methods, training miners on using those methods, and raising environmental awareness among the miners.

## Data Availability

Data can be provided on demand.
